# Stabilization and Solidification of Beryllium Waste: Influence of the Cement Composition on the Corrosion of Be Metal

**DOI:** 10.3390/ma17225401

**Published:** 2024-11-05

**Authors:** Richard Laflotte, Céline Cau Dit Coumes, Jérémy Haas, David Rodrigues, Céline Cannes, Sylvie Delpech, Murielle Rivenet

**Affiliations:** 1 CEA, DES, ISEC, DPME, SEME, LFCM, Université de Montpellier, 30207 Bagnols-sur-Cèze, France; celine.cau-dit-coumes@cea.fr (C.C.D.C.);; 2 IJCLab, CNRS/IN2P3, Université Paris-Saclay, 91405 Orsay, France; david.rodrigues@ijclab.in2p3.fr (D.R.); celine.cannes@ijclab.in2p3.fr (C.C.); sylvie.delpech@ijclab.in2p3.fr (S.D.); 3 Univ. Lille, CNRS, Centrale Lille, Univ. Artois, UMR 8181, UCCS—Unité de Catalyse et Chimie du Solide, F-59000 Lille, France

**Keywords:** beryllium, cement, suspension, corrosion, electrochemical methods, waste conditioning

## Abstract

Beryllium metal is used as neutron moderator and reflector or multiplier in certain types of fission or fusion reactors. Dismantling of these reactors will produce radioactive beryllium waste, classified as low- or intermediate-level waste, that will need to be stabilised and solidified before being sent to disposal. The cementation process is under consideration because it may offer a good compromise between simplicity of implementation, cost, and quality of the final cemented wasteform. Nevertheless, knowledge of the corrosion behaviour of Be metal in a cement-based matrix is still limited, partly due to the high toxicity of Be that complicates testing. This study thus investigates Be corrosion in cement suspensions using potentiometry, voltammetry, and electrochemical impedance spectroscopy. Among the five different investigated systems (Portland cement blended without or with 40 wt.% silica fume, calcium sulfoaluminate clinker blended without or with 15% anhydrite, and calcium aluminate cement), Portland cement blended with 40% silica fume and calcium sulfoaluminate cement comprising 15% anhydrite are the most effective in mitigating beryllium corrosion. They allow reduction in the corrosion current by factors of 4 and 50, respectively, as compared to Portland cement.

## 1. Introduction

Beryllium is used in the nuclear industry in certain types of reactors as a neutron moderator and reflector for its low neutron-capture and high neutron-scattering cross-sections [1]. Furthermore, its neutron-multiplying properties make it a material of interest for nuclear fusion applications to ensure tritium self-sufficiency. For instance, beryllium has been used in the form of blankets as a plasma-facing component inside the vacuum chamber of the experimental UNITOR, ISX-B, and JET tokamak reactors [2,3,4,5,6] and is under consideration for a future ITER fusion facility [7,8]. Under neutron irradiation in fusion or fission reactors, beryllium is contaminated with short- and long-lived activation products (such as ^3^H, ^10^B or ^14^C, ^41^Ca, ^60^Co …) due to the presence of impurities [7,8,9,10]. It thus becomes a low-level or intermediate-level radioactive waste once the reactors are dismantled. A management route under investigation is its immobilisation into a cement-based matrix before storage.

### 1.1. Reactivity of Be Metal in a Cement-Based Matrix

**Figure 1:**
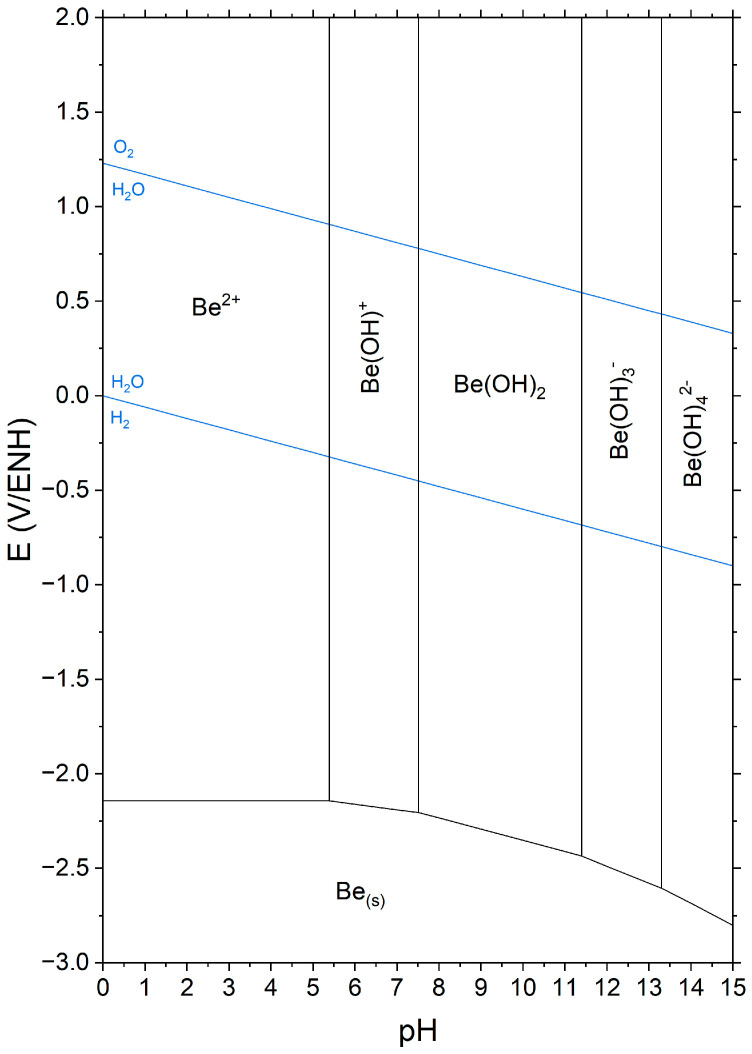
E-pH diagram of beryllium established from the data of Cevirim-Papaioannou et al. [13] ([Be(II)] = 10^−6^ mol/L, T = 25 °C).

Hardened cementitious materials are porous media composed of solid phases and, for usual formulation parameters and curing conditions, of a solution located within the pore network. The chemistry of this pore solution depends on the solubilities of the phases present. When Be metal is encapsulated in a cement matrix, it can be corroded by the pore solution, with concomitant production of dihydrogen that may be detrimental to the safe storage of cemented waste packages. The potential-pH diagram of beryllium, first established by Pourbaix in 1963 [11], and recently updated by Bouhier et al. [12] using revisited thermodynamic data reported in the literature [13,14,15], is presented in Figure 1. It shows that Be metal is not thermodynamically stable in an aqueous environment. It is oxidised into Be^2+^ cations in acidic medium (Equation (1)), into Be(OH)_2_ (8 < pH < 13 for a 10^−6^ mol/L Be(II) concentration (Equation (2)), and Be(OH)_3_^−^ and Be(OH)_4_^2−^ oxyanions in alkaline conditions (Equations (3) and (4)).

(1) $${Be}_{\left(s\right)}+2H^{+}\to {{Be}^{2+}}_{\left(aq\right)}+{H_{2}}_{\left(g\right)}$$

(2) $${Be}_{\left(s\right)}+2H_{2}O\to {{Be\left(OH\right)}_{2(\alpha )}}_{\left(s\right)}+{H_{2}}_{\left(g\right)}$$

(3) $${Be}_{\left(s\right)}+{HO}^{-}+2H_{2}O\to {{{Be\left(OH\right)}_{3}}^{-}}_{\left(aq\right)}+{H_{2}}_{\left(g\right)}$$

(4) $${Be}_{\left(s\right)}+{2HO}^{-}+2H_{2}O\to {{{Be\left(OH\right)}_{4}}^{2-}}_{\left(aq\right)}+{H_{2}}_{\left(g\right)}$$

Passivation of the metal may only be expected in the stability domain of Be(OH)_2_, provided that a dense protective layer is formed at the solution/metal interface. Previous studies carried out with aqueous solutions of variable pH (from 2 to 15) [16,17] or various cement pastes [12] confirmed that the corrosion rate of Be is minimal at pH close to 12 (Table 1).

### 1.2. Selection of Cements

The objective of this work was to complement previous studies by investigating more specifically the chemical compatibility of Be metal with cements producing a pore-solution pH around 12, which seems to be optimal for minimising Be corrosion and mitigating the release of dihydrogen. 

Portland cement, which is widely used for radwaste conditioning, produces a highly alkaline pore solution, with a pH above 13 that exceeds the target value [18]. This high pH is mainly controlled by the dissolution of alkalis (Na_2_O, K_2_O). When these alkalis are leached out, the pH still remains buffered at 12.5 due to the dissolution of portlandite [19,20,21]. However, blending Portland cement with silica-rich pozzolanic materials such as silica fume makes it possible to decrease the pH to below 12.5 [22,23,24,25,26,27,28]: portlandite is indeed depleted by the pozzolanic reaction, and calcium silicate hydrates (C-S-H) with low Ca/Si ratio and reduced equilibrium pH are formed. For instance, pore-solution pH values ranging from 11.3 to 12.2 were reported for cement pastes comprising 60% Portland cement and 40% silica fume after one year of curing in sealed bags at ambient temperature. The dispersion of data mainly results from different degrees of reaction of the silica fume [23,26,29].

Alternatively, binders other than calcium silicate cements, involving different hydration reactions, may produce less alkaline pore solutions than Portland cement. This is the case for calcium aluminate cement of the Fondu^®^ type, mainly comprising calcium aluminate phases (CA, C_4_AF, and C_12_A_7_ using shorthand cementation notations (C = CaO, S = SiO_2_, $\bar{S}$ = SO_3_, A = Al_2_O_3_, F = Fe_2_O_3_, H = H_2_O)), which leads to a pore-solution pH ranging from 11.4 to 12.5 [30]. Calcium aluminate phases are hydrated into metastable hydrates (CAH_10_ and C_2_AH_8_), katoite (C_3_AH_6_), and aluminium hydroxide (AH_3_). Metastable phases are then gradually converted into C_3_AH_6_ and poorly crystallised AH_3_. 

Calcium sulfoaluminate cements are other potential candidates for beryllium passivation. Their clinker is mainly composed of ye’elimite (C_4_A_3_$\bar{S}$), belite (C_2_S), and an Al-rich ferrite phase [31] that are usually interground with a calcium sulfate source (gypsum or anhydrite). Their hydration is usually fast and strongly depends on the amount and reactivity of the calcium sulfate source [32]. It starts with the initial precipitation of ettringite and aluminium hydroxide, followed by the precipitation of calcium monosulfoaluminate hydrate once the calcium sulfate has been depleted [33,34,35,36]. Belite has a slower hydration rate [37] and mainly yields strätlingite, siliceous hydrogarnet, and/or C-S-H depending on its initial content in the clinker. The pore-solution pH of calcium sulfoaluminate cement pastes is set at 10.5–11, as long as ettringite is formed, and then increases to 11.5–12.5 once the calcium sulfate source is exhausted and calcium monosulfoaluminate hydrate is the main hydrated phase formed [36,38].

Finally, based on this literature review, five cements were selected for further investigation in this work: Portland cement (reference), Portland cement (60 wt.%) blended with silica fume (40 wt.%), Fondu^®^ cement, and calcium sulfoaluminate clinker blended without or with 15 wt.% anhydrite (to promote the formation of calcium monosulfoaluminate hydrate and ettringite, respectively).

### 1.3. Experimental Approach

In a cement paste, the mixing water is gradually consumed by the hydration reactions of the binder. When studying the corrosion of beryllium in cement paste, the question of a possible limitation of corrosion by lack of water therefore arises. Furthermore, the progressive evolution of the chemistry of the cement paste towards a state of equilibrium leads to a variation in the pH and chemical composition of the pore solution. A study using a synthetic solution with the same composition as the pore solution of the cement pastes has the main disadvantage of lacking a reservoir to regulate the composition of the solution. On the contrary, in a cement paste, the composition of the pore solution is controlled by dissolution equilibria of the hydrates.

In this work, it was thus decided to investigate corrosion of Be metal using cement suspensions, which presents several advantages:

- The pH and composition of the solution are controlled by the dissolution of cement hydrates, and their evolution is thus limited during the test;
- All species present in the pore solution are taken into account, whereas the composition of synthetic solutions is generally simplified;
- Corrosion of Be cannot be limited by a lack of water, contrary to cement pastes.

Different methods may be used to monitor the corrosion of Be in cement suspensions. Results obtained by gravimetry (measurement of the weight loss of the metal as a function of time) or by analysing the solution composition (dosage of aqueous Be species released in solution by ICP-AES) may be biased by the precipitation of an oxide layer at the metal/solution interface. Beryllium toxicity and sputtering also prevent implementing any advanced observation technique (IR, Raman, SEM/EDX, or SIMS) or any preparation shaping techniques (cutting, polishing …) without using a glovebox. Corrosion monitoring of beryllium can also be achieved using electrochemical techniques. The corrosion current can be measured by voltammetry. This technique is not well adapted to cement pastes due to their high electrolyte resistance, but it can be implemented in cement suspensions. However, this technique implies polarising the electrode, which may impact the electrolyte/metal interface. To avoid imposing large potential variations on the beryllium electrode, impedance spectroscopy, which allows one to work at low potential amplitudes (~10 mV) around the open circuit potential (OCP), is a more suitable method to collect data on beryllium corrosion. Finally, measuring dihydrogen production due to water reduction by gas chromatography can provide information on the corrosion rate of Be, but this method may be biased if other non-aqueous oxidation routes of Be (for instance by dissolved O_2_) are involved.

In this work, the focus was placed on potentiometric and electrochemical methods, which were revealed to be successful for investigating the corrosion of other metals in cementitious environments [16,39,40,41,42]: monitoring the OCP evolution of the metal studied with time, measuring the current intensity corresponding to Be oxidation in the different cement suspensions by cyclic voltammetry, and recording electrochemical impedance spectra at the OCP. This latter method can be easily repeated with time and makes it possible to investigate the progress of corrosion. Furthermore, quantitative analysis of the spectra can provide information on the corrosion mechanisms and corrosion current.

## 2. Materials and Methods

### 2.1. Raw Materials

Three commercial binders were used in this work: Portland cement (PC 52.5 PM ES CP2—Lafarge Holcim, Le Teil, France) (PC), calcium aluminate cement (Fondu^®^, Imerys Aluminates, Dunkerque, France) (CAC), and calcium sulfoaluminate (CSA) clinker (Alpenat-Up CK—Vicat, Saint Egrève, France). Portland cement was used pure (reference) or blended with silica fume (Condensil S95 DM, Chambéry, France). Similarly, the calcium sulfoaluminate clinker was used as supplied or mixed with anhydrite (Vicat). The main characteristics of the raw materials, based on data measured experimentally (in this work or in References [32,43,44]) and provided by the supplier, are summarised in Table 2.

### 2.2. Preparation of Cement Suspensions

Cement pastes were first prepared using Portland cement (PC), Portland cement blended with silica fume (SF) (B40: 60% PC, 40% SF), Fondu cement (CAC), calcium sulfoaluminate clinker (CSA0), and calcium sulfoaluminate clinker blended with 15% anhydrite (CSA15). Their water content was adjusted to obtain workable materials without any bleeding, resulting in water-to-cement ratios of 0.4 (PC, CAC) or 0.5 (B40, CSA0, CSA15). The pastes were cured at 20 °C and 95% R.H. for 34 days (CSA0) to 54 days (PC, B40, CAC, CSA15) and then ground by hand to a particle size below 2 mm. Suspensions were prepared by mixing demineralised water with the ground paste samples at a liquid-to-solid ratio of 5 mL/g. This latter resulted from a compromise: the amount of water was maintained as low as possible to limit the dilution of alkalis present in the cement pastes, while allowing immersion of the electrodes without any contact with the solid particles during the measurements. The suspensions were maintained under stirring in tightly sealed reactors to limit carbonation. Stationary pH was reached after 24 h and ranged from 11.7 (CSA15) to 12.7 (PC).

### 2.3. Electrochemical Cells

**Figure 2:**
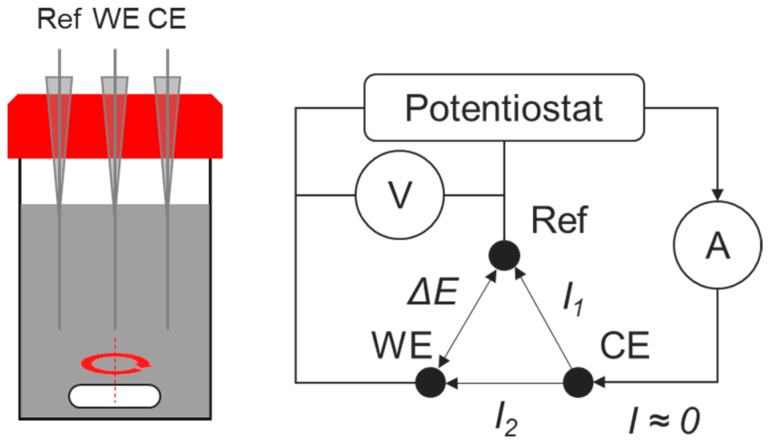
Electrochemical cell.

Electrochemical cells, similar to those used by Lahalle [45], Poras [41], and Bouhier [12], had a volume of 100 mL and comprised 3 electrodes: the working electrode (WE) in beryllium or platinum, the “reference” (Ref), and counter electrodes (CEs) in platinum (Figure 2). The “reference” electrode in platinum was in fact a pseudo-reference electrode since its potential depended on the pH of the system. A correlation, established by Bouhier [46] and giving the potential of the platinum electrode versus that of the normal hydrogen electrode (NHE) as a function of pH, was used to recalculate the measured potential values versus the NHE. Note that commercial reference electrodes such as the Ag/AgCl electrode were not selected to avoid (i) clogging of the glass frit by the cement particles and (ii) diffusion of electrolyte ions (such as chlorides) into the cement suspension, which would modify its composition and could influence Be corrosion. The platinum and beryllium wires (1 mm and 1.2 mm by diameter, respectively, supplied by Alfa Aesar, Karlsruhe, Germany and Goodfellow, Lille, France) had purities of 99.95% and 99.7%, respectively. Before use, Pt was cleaned with the flame of a burner, rinsed with ethanol, and then rinsed with water. Be electrodes were rinsed with HCl (0.1 mol L^−1^), then with deionised water until their colour turned to light grey, and finally with ethanol before being dried with tissue paper. The electrodes were fixed to the lid of the reactor using plastic cones and Araldite^®^ glue, at a distance of c.a. 2 cm between each other. The immersed length of Be and Pt wires in the suspensions was about 3 cm, corresponding to a reactive surface of about 1.1 cm^2^ for Be electrodes and 0.9 cm^2^ for Pt electrodes. The reactors were first filled with 100 mL of cement suspension, and the lid with the electrodes was tightly sealed. All the suspensions, except the CSA15 one (due to a technical issue), were maintained under magnetic stirring between two electrochemical measurements. Given the chemical toxicity of beryllium, the experiments were performed in a glovebox at ambient temperature under air atmosphere.

### 2.4. Electrochemical Techniques

Electrochemical measurements were performed using VersaSTAT 4 potentiostat (Ametek, Wayne, NJ, USA) piloted with VersaStudio version 2.63.3 software. Three techniques were implemented to characterise the corrosion of beryllium in the cement suspensions. Potentiometry was performed to measure the open circuit potential (OCP) (or potential at zero current) between the working and reference electrodes. Cyclic voltammetry was performed at a scan rate of 20 mV/S to determine the cathodic limit of the water stability domain relative to the platinum electrode for each suspension, as well as the oxidation current of beryllium. The voltammetry diagrams were corrected from the ohmic drop to take into account the electrolyte resistivity of the suspensions.

Electrochemical impedance spectroscopy (EIS) was performed at the OCP to avoid polarising the electrode, which may modify the Be/electrolyte interface. A low amplitude (10 mV) sinusoidal potential variation ΔE was superimposed to the OCP between working and reference electrodes (Equation (5)), over a decreasing frequency (f) range (from 10^6^ to 10^−1^ Hz), with 10 points per decade. The resulting sinusoidal current, ΔI superimposed to the stationary current I flowing from the counter-electrode to the working electrode and phase-shifted by an angle ϕ with respect to the potential, was measured (Equation (6)).

(5) $$E\left(t\right)=OCP+\Delta E\cdot \sin\left(2\pi ft\right)$$

(6) $$I\left(t\right)=I+\Delta I\cdot \sin\left(2\pi ft+\phi \right)$$

The electrochemical impedance was defined as the ratio between the Laplace transforms of E(t) and I(t) (Equation (7)).

(7) $$Z=\frac{L_{t}\left[E\left(t\right)\right]}{L_{t}\left[I\left(t\right)\right]}=\frac{\bar{\Delta E}}{\bar{\Delta I}}=Z_{real}+iZ_{imag}$$

Impedance Z included a real part (Z_real_) and an imaginary part (Z_imag_) that made it possible to draw Nyquist and Bode diagrams by plotting respectively—Z_imag_ vs. Z_real_, log|Z| and ϕ vs. log(f) (|Z| standing for the impedance modulus and ϕ its phase). 

### 2.5. Analysis of Suspensions

The pH values of the suspensions were measured using a pH electrode (Mettler Toledo InLab^®^ Micro-Pro-ISM pH 0–14, Zurich, Switzerland) previously calibrated with IUPAC standards at 9.2 and 12.5 (25 °C). At the end of the experiments, the suspensions were filtered at 0.2 µm under vacuum on a Buchner funnel. The collected solutions were acidified and diluted with a 2 wt.% nitric acid solution before analysis by ICP-AES (Thermo Fischer ICAP 6300 Duo, Waltham, MA, USA) previously calibrated with external standards (solutions of Ca, Al, Si, Na, K, and S). The experimental pH determinations were compared to pH values calculated with CemGEMS version 0.8.1 [47] at thermodynamic equilibrium, thus assuming full hydration of cement.

## 3. Results and Discussion

### 3.1. Characterisation of Cement Suspensions

**Figure 3:**
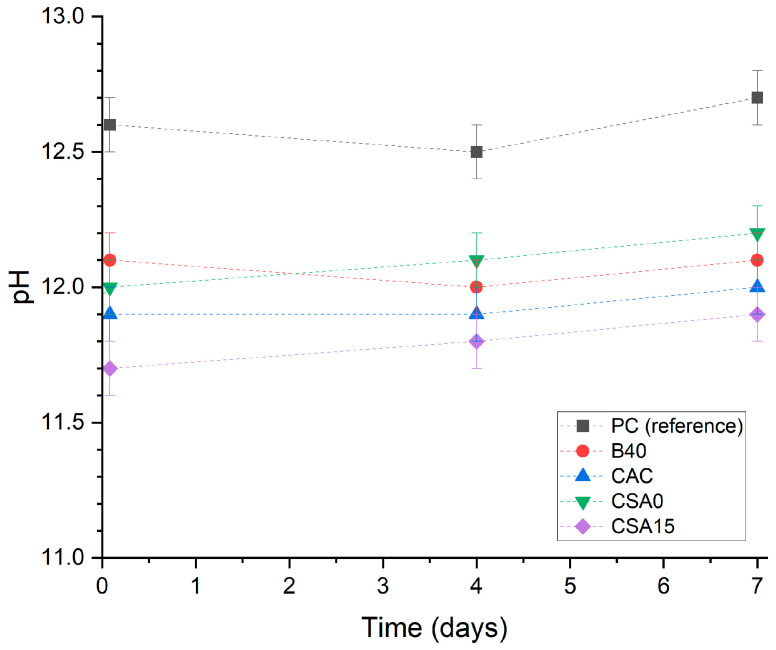
pH evolution over the measurement period.

The pH of the suspensions did not show any significant evolution over the duration of the study (Figure 3). As expected, Portland cement led to the highest pH, slightly above 12.6 due to slight dilution of alkalis. Inversely, calcium sulfoaluminate clinker blended with 15% anhydrite yielded a pH slightly below 12. The measured pHs were in reasonably good agreement with those calculated using CemGEMS at thermodynamic equilibrium, except for suspensions B40 and CSA15, which presented values, respectively, above and below the calculated ones (Table 3). These results suggest incomplete hydration of cements B40 and CSA15. In the first case, the pH is indeed expected to decrease when the pozzolanic reaction progresses. On the contrary, in the second case, the depletion of calcium sulfate should lead to a pH increase. The concentrations of the main dissolved species determined by ICP-AES are summarised in Table 3. The PC solution showed the highest Ca, Na, and K concentrations. An unexpectedly high sulfate concentration was measured in suspension B40, which could have resulted from impurities present in the silica fume (SO_3_ content of 0.24 wt.%). The aluminate concentration was higher in calcium aluminate and sulfoaluminate binders.

### 3.2. OCP Measurement

The OCP, which characterises the redox reactions taking place at the Be electrode, was measured after increasing immersion times in the cement suspensions. The aqueous corrosion of Be could be simply assessed by comparing the OCP value to the reduction potential of water. The latter was previously determined for each suspension using voltammetry on a Pt working electrode (Equation (8)).

(8) $${2H}_{2}O+{2e}^{-}={H_{2}}_{\left(g\right)}+{2HO}^{-}$$

The H_2_O/H_2_ redox potentials determined from the voltamograms (Table 4) were close to thermodynamic values calculated using the Nernst equation (Equation (9)), at P(H_2_) = 1 atm, with R the molar gas constant and F the Faraday constant.

(9) $$E\left(H^{+}/H_{2}\right)=E^{0}\left(H^{+}/H_{2}\right)-\frac{2.3\cdot R\cdot T}{F}\cdot pH$$

**Figure 4:**
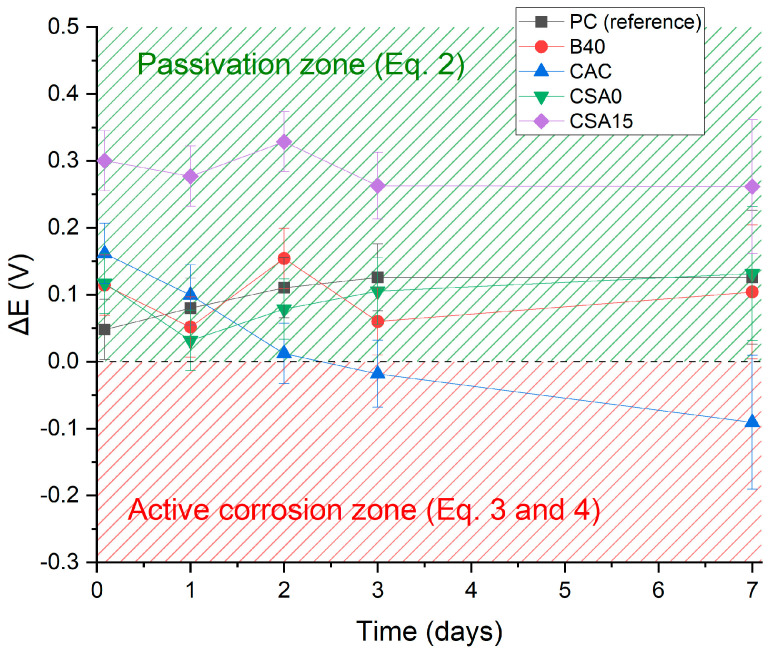
Evolution of the difference between the OCP value and the H_2_O/H_2_ redox potential in the investigated cement suspensions.

In case of aqueous corrosion of beryllium, the OCP should be below the cathodic limit of water stability, and the lower the OCP with respect to this limit, the stronger the corrosion of beryllium. Figure 4 plots the evolution of ΔE, the difference between Be OCP and the water reduction potential (E(H_2_O/H_2_)) in the different cement suspensions. Positive ΔE values were obtained with every suspension at the beginning of the measurements, meaning that Be tended to passivate in all systems (Equation (2)). The CSA15 suspension led to the highest ΔE values, suggesting a better passivation of Be in such an environment. However, with the CAC system, ΔE tended to decrease to values close to 0 after 2 days. This suggests that beryllium may tend to depassivate with time in this medium. 

### 3.3. Voltammetry

**Figure 5:**
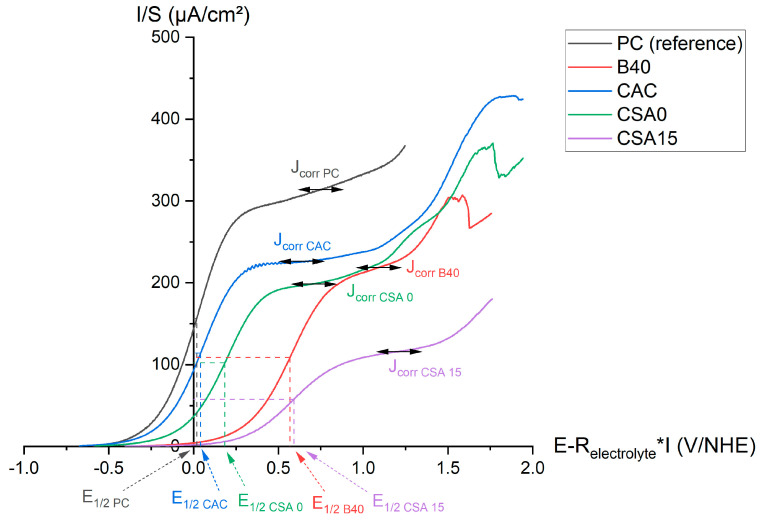
Voltammetry curves (potential corrected from the ohmic drop) of Be in the cement suspensions after 3 days of immersion.

Figure 5 shows the voltammetry curves obtained on the beryllium electrode after 3 days of immersion in the different cement suspensions. The current intensity was normalised with respect to the surface area of Be in contact with the suspensions, and the potential was corrected by the ohmic drop using the electrolyte resistance determined by EIS (see Section 3.5). When the potential was scanned from the OCP to 2 V, an oxidation wave was observed. The current started to increase and reached a plateau, indicating a diffusion-limiting step through a passivation layer formed at the metal/solution interface. At higher potentials, Be was depassivated, and the current showed an exponential increase. The current at the plateau, corresponding to the corrosion current of Be, varied with the composition of the suspension (Table 5). The lower its value, the more passivated the metal. The oxidation half-wave potential (E_1/2_), i.e., the potential at the half-height of the diffusion plateau, also helped with discriminating between the different systems (Table 5): the higher the potential, the more difficult the corrosion of beryllium since a higher voltage was needed for metal oxidation.

The highest I_corr_/S value was measured in PC suspension, and the lowest was measured in CSA15 suspension. E_1/2_ values were significantly higher in CSA15 and B40, indicating that beryllium was more difficult to corrode in these suspensions. In suspension B40, however, the corrosion current was close to those measured in CAC and CSA0 suspensions, despite the higher E_1/2_ value. This suggests that Be was less passivated in B40 than in CSA15 suspension.

### 3.4. Electrochemical Impedance Spectroscopy—Qualitative Approach

#### 3.4.1. Suspension Contribution

**Figure 6:**
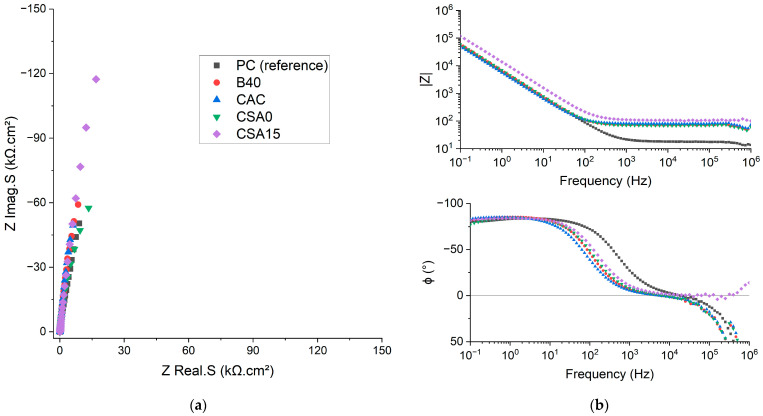
Nyquist (**a**) and Bode (**b**) (impedance modulus on the upper right, impedance phase on the lower right) diagrams recorded on a platinum electrode in the cement suspensions after 3 days of immersion.

Before investigating Be corrosion, it was first necessary to assess the electrochemical contribution of the cement suspension to the impedance. To this end, EIS measurements were carried out using a platinum working electrode, which was considered as inert with respect to corrosion. The Nyquist diagrams (normalised by the electrode surface area in contact with the suspension) and Bode diagrams obtained after 3 days of immersion of Pt in the different cement suspensions are given in Figure 6.

While all Bode diagrams presented a high impedance modulus at 0.1 Hz, a significant difference was observed at high frequencies, where the contribution of the suspensions was observed. The impedance modulus of the PC suspension levelled off at ≈20 Ω.cm^2^, which is one order of magnitude smaller than the values obtained for the other suspensions. This means that the PC suspension had a lower electrolyte resistance, or higher electrical conductivity, than the others. This result is consistent with the ionic conductivity measurements presented in Table 6. The hydroxide ions, more concentrated and with a high mobility, contributed to enhance the ionic conductivity in the PC system.

#### 3.4.2. Be Corrosion

**Figure 7:**
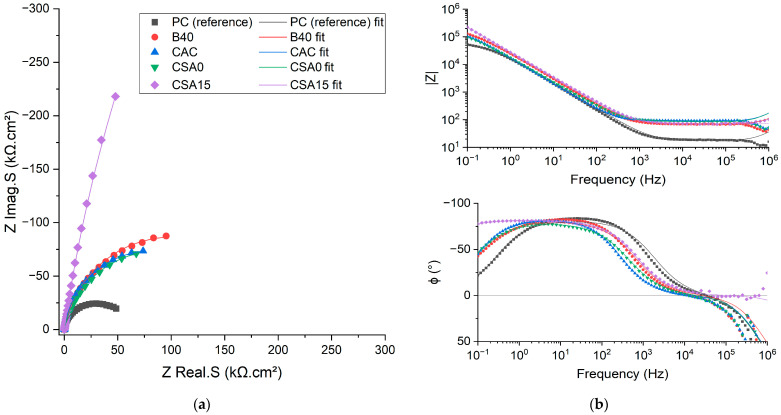
Nyquist (**a**) and Bode (**b**) diagrams of Be electrode in the cement suspensions after 3 days of immersion (experimental (points) and modelled (lines) results).

**Figure 8:**
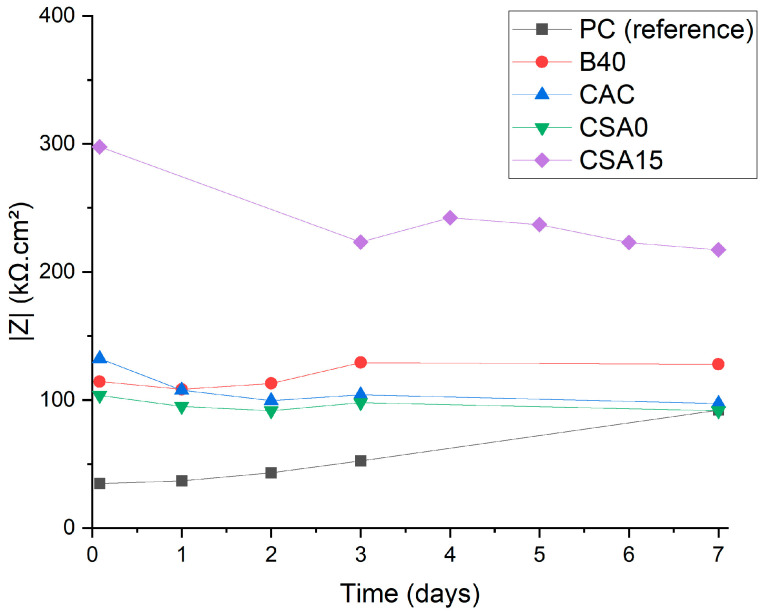
Evolution of the impedance modulus at 0.1 Hz recorded on the Be electrode as a function of the immersion time.

The impedance spectra recorded on the Be electrode after 3 d of immersion are presented in Figure 7. The spectra obtained after 1 day (d) and 7 d of immersion are given in Appendix A. Nyquist diagrams showed a capacitive loop, with a diameter that increased when corrosion decreased. The PC suspension yielded the lowest diameter and, thus, the strongest corrosion of Be. Conversely, the loop diameter was maximal in CSA15 suspension, indicating minimal corrosion of Be. When the Be immersion time increased, the capacitive loop diameter increased notably in B40 suspension, and to a smaller extent in CSA0 and PC suspensions, whereas it tended to decrease in CAC suspension, which is consistent with the OCP evolution reported Section 3.2. Comparing the impedance modulus at low frequency (0.1 Hz) also enabled qualitative assessment of the extent of corrosion (Figure 8). CSA15 suspension led to a significantly higher impedance modulus at 0.1 Hz, which means that beryllium was less corroded in this suspension. On the contrary, PC suspension produced the lowest impedance modulus, suggesting, therefore, the strongest corrosion of beryllium in this environment.

Based on the results obtained after 3 days of immersion, it is possible to classify the different systems in order of increasing corrosion of Be: CSA15 < B40, CAC, and CSA0 < PC.

This classification is consistent with the voltammetry results. Indeed, PC and CSA15 suspensions led to the highest and lowest corrosion currents, respectively. Similarly, the highest oxidation half-wave potential values were obtained in CSA15 and B40 suspensions. Comparing the OCP to the reduction potential of water also confirmed the good passivation of Be in the CSA15 suspension. This method also suggested the progressive depassivation of Be in the CAC suspension, which needs to be confirmed by determining the corrosion current and its evolution with time.

### 3.5. Electrochemical Impedance Spectroscopy—Quantitative Approach

In a second stage, quantitative analysis of the impedance spectra was carried out. A three-step mechanism (Equations (10)–(12)), previously developed by several authors [42,48,49,50,51,52,53,54], was postulated to describe beryllium corrosion.

1. Metal Be oxidation into Be(II):

(10) $${Be}_{\left(s\right)}+\underset{\Gamma \left(1-\theta \right)}{s}\overset{k_{an}}{\to }\underset{\Gamma \theta }{{\mathrm{Be}}^{2+},s}+2e^{-}$$

2. Reduction of water:

(11) $$2H_{2}O+2e^{-}\overset{k_{cath}}{\to }H_{2}+2{HO}^{-}$$

3. Desorption (renewal of electroactive sites):

(12) $$\underset{\Gamma \theta }{{Be}^{2+},s}\overset{K}{\to }{\mathrm{Be}}^{2+}+\underset{\Gamma \left(1-\theta \right)}{s}$$

**Figure 9:**
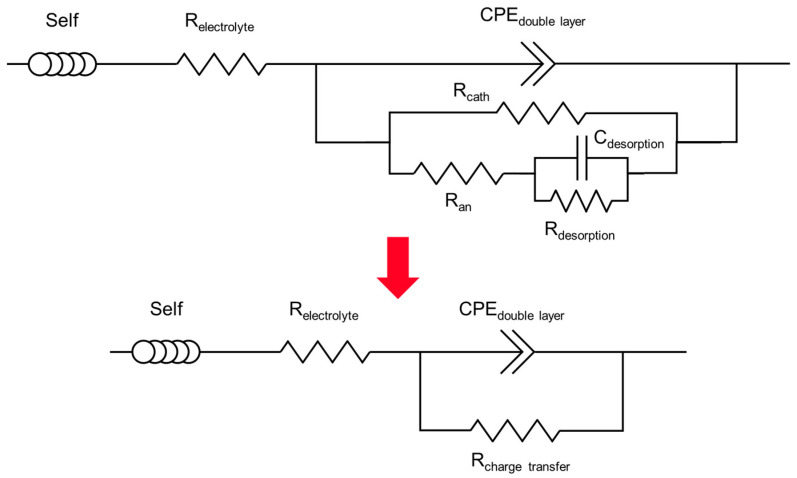
Electrical circuit used to fit the EIS spectra.

An equivalent electrical circuit was associated to this mechanism, where all the electrical parameters were expressed as a function of the kinetic parameters (Figure 9). The calculations are detailed in Appendix B.

To model the impedance spectra, it was also necessary to take into account the contribution of the cement suspension, simply described by a resistance (R_electrolyte_ in Figure 9) accounting for its intrinsic electrical resistance. An inductance, denoted as “Self” in Figure 9, was added to model the self-induction effect observed at very high frequencies (f ≈ 10^6^ Hz) on Nyquist and Bode diagrams due to electrical disturbances. Finally, since the electrodes were plunged in cement suspensions, an electrical double layer of ions formed at the electrode/electrolyte interface. The electrochemical impedance of the double layer was modelled by a constant phase element (CPE_double layer_) [55,56] in parallel with the charge transfer impedance. As shown in previous studies [55,56,57], the constant phase element makes it possible to reflect the non-ideality of the capacitance due to surface inhomogeneities of the electrode (roughness, impurities, variations in coating layer…). It includes two terms (Equation (13)): capacitance C_double layer_, expressed in F/cm^2^, and coefficient α, varying between 0 and 1 and depicting its non-ideality (α = 1: ideal capacitor; α = 0: resistor, 0.85 ≤ α ≤ 1 to describe heterogeneities at a metal/solution interface).

(13) $$Z_{CPE}=\frac{1}{C_{\mathrm{double}\mathrm{layer}}\cdot {\left(j\cdot \omega \right)}^{\alpha }}$$

The charge transfer impedance was developed into two parallel branches modelling water reduction at the cathode and beryllium oxidation at the anode. A R//C circuit was added in the anodic branch to model the desorption step (Figure 9). Nevertheless, Cannes et al. [16] observed that, in NaOH solutions at pH ≥ 11.8, the capacitance branch linked to the desorption process (C_desorption_) could be neglected due to passivation of the electrode. The faradaic impedance then simplified into 3 resistances, which, finally, were equivalent to a pure faradaic resistance R_charge transfer_.

**Figure 10:**
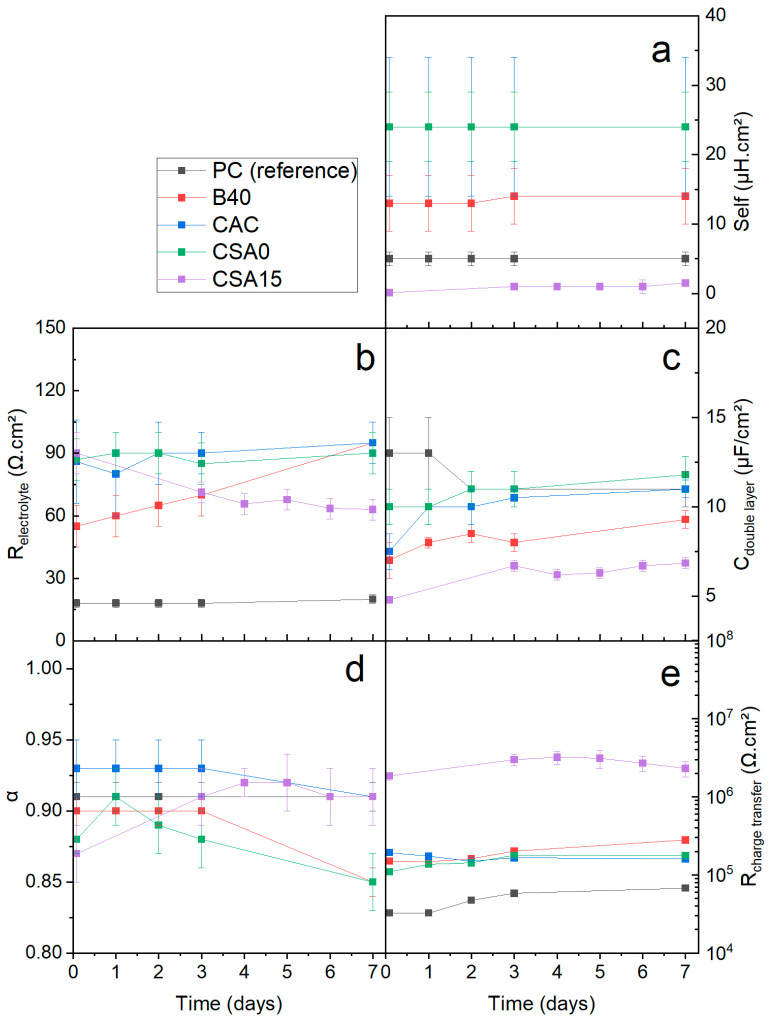
Influence of the type of cement suspension on the electrical parameters of the equivalent circuit. (**a**) self-inductance (**b**) electrolyte resistance (**c**) double layer capacitance (**d**) double layer CPE α coefficient (**e**) charge transfer resistance.

**Figure A1:**
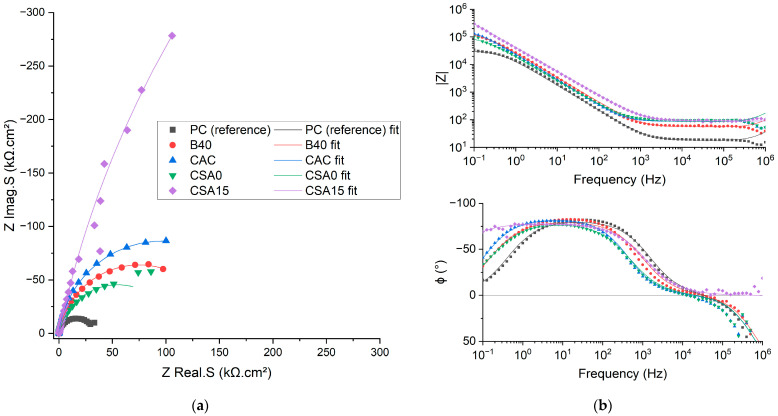
Nyquist (**a**) and Bode (**b**) diagrams recorded for Be after 1 d of immersion in the cement suspensions (experimental (dots) and modelled (lines) results).

**Figure A2:**
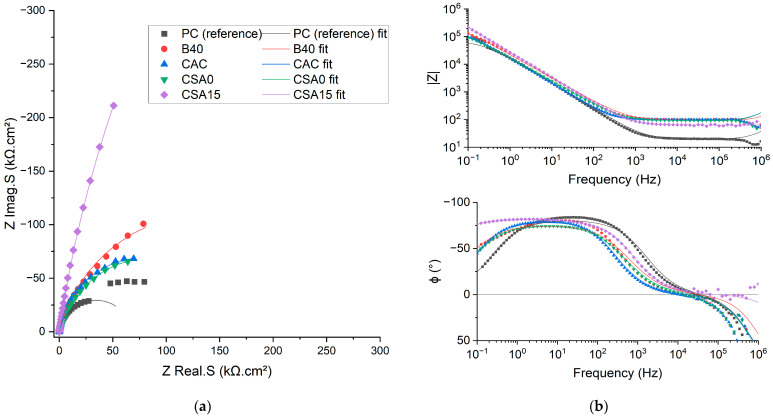
Nyquist (**a**) and Bode (**b**) diagrams recorded for Be after 7 d of immersion in the cement suspensions (experimental (dots) and modelled (lines) results).

The electrical parameters were adjusted by fitting the experimental impedance spectra using Zview^®^ version 3.5g software [58]. The equivalent electrical circuit enabled obtaining fits of good quality for all investigated systems (Figure 7, and Figure A1 and Figure A2 in Appendix A). The optimised values of the electrical parameters are summarised in Table A1 (Appendix C), and Figure 10 compares their evolution for the different cement suspensions.

Regardless of the cement suspension, the electrolyte resistance values were consistent with those inferred from the EIS spectra recorded on a Pt working electrode and given by the plateau values of the impedance modulus at high frequency in Figure 6. Furthermore, the electrolyte resistance is linked to its electrical conductivity (Equation (14)).

(14) $$\gamma =\frac{k}{R_{electrolyte}}$$

where k is a geometric parameter depending on the distance between the electrodes and on their surface area. This parameter was determined by measuring the electrolyte resistance of standard KCl solutions of known ionic conductivities (1.413 mS/cm and 12.88 mS/cm at 25 °C) by EIS using the same electrochemical cell geometry as for the cement suspensions. The k factor was equal to 207 cm^−1^. The ionic conductivities calculated using Equation (14) were in good agreement with those measured experimentally using a conductivity sensor (Table 6). A decrease in the ionic conductivity in suspension B40 was noticed over the measurement period (γ = 3.5 mS/cm after 1 d, γ = 2.7 mS/cm after 7 d), which was consistent with the increasing R_electrolyte_ observed by EIS.

R_electrolyte_ showed little change over the duration of the study for PC, CAC, and CSA0 suspensions (Figure 10b). On the contrary, the resistance of B40 suspension tended to increase slightly, while that of CSA15 suspension decreased over time. Such evolutions were well correlated to the pH variations observed in these suspensions. In the case of B40 suspension, the decrease in pH could be explained by the progress of the pozzolanic reaction between silica fume and the cement phases, promoted by grinding of the cement paste, addition of water to prepare the suspensions, and abrasion occurring during stirring of the suspension. CSA15 suspension showed initially the highest resistance, but this later tended to decrease and finally stabilised between 60 and 70 Ω.cm^2^. This decrease could be due to the bad stirring of this suspension, possibly leading to local concentration heterogeneities. Another hypothesis could be a change in the composition of the solution (even if its pH did not evolve significantly) due to the restart of cement hydration. As expected, the PC suspension, with the highest pH and conductivity, showed the lowest electrolytic resistance. 

The self-induction parameter (Figure 10a) varied from one suspension to another but remained constant over the whole measurement period. The double-layer capacitance showed little variation over time (Figure 10c) and from one system to another (values between 5 (CSA15) and 13 µF/cm^2^ (PC)). For comparison, the C_double layer_ values of Be in aqueous sodium hydroxide solutions, having a pH in the range 11.8–14, were close to 40 µF/cm^2^ [16]. The non-ideality of the electrode/solution interface resulted in α values slightly less than 1 (comprising values between 0.85 and 0.95) (Figure 10d) [59].

### 3.6. Corrosion Rate of Be and Dihydrogen Production

The corrosion current of Be in the various cement suspensions was calculated from the resistance to charge transfer (Equation (15)). The measurements being performed at the OCP, the anodic current, i.e., the Be oxidation current, was equal to the opposite of the cathodic current. I_corr_ could be expressed as follows:

(15) $$I_{corr}=\frac{R\cdot T}{n_{e}{\cdot \alpha }_{cath}\cdot F{\cdot R}_{\mathrm{charge}\mathrm{transfer}}}$$

with R the molar gas constant (in J·mol^−1^·K^−1^), T the temperature (in K), F the Faraday constant (in C·mol^−1^), and α_cath_ the charge transfer coefficient for water reduction, equal to 0.5 [46].

(16) $$J_{corr}=\frac{I_{corr}}{S}$$

**Figure 11:**
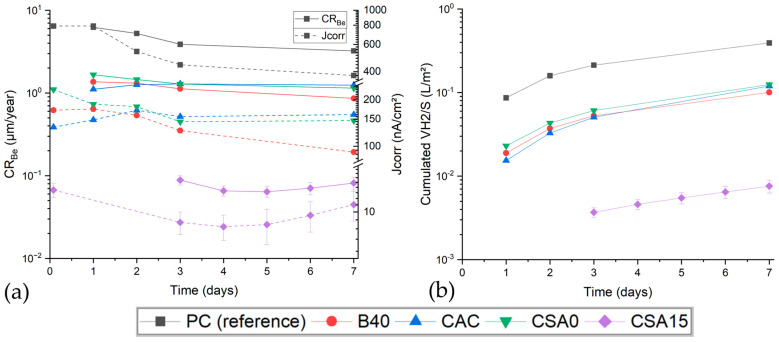
Influence of the type of cement on the Be corrosion rate and current (**a**) and on the cumulative volume of H_2_ (normalised by the electrode surface area) (**b**).

Evolution of J_corr_, the corrosion current normalised with respect to the initial electrode surface area (Equation (16)), is shown in Figure 11a for the different cement suspensions. The Be corrosion current tended to decrease with time, except for with the CAC suspension. This confirms the particular behaviour of this system, previously evidenced by the OCP measurements. Furthermore, J_corr_ was reduced by at least one order of magnitude in CSA15 with respect to other suspensions, showing a strong passivation of beryllium. J_corr_ was then integrated over time, which was related to the mol number of beryllium oxidised using the Coulomb’s law (Equation (17)).

(17) $$Q_{/S}=\int_{t}J_{corr}=n_{e}{\cdot n\left(H_{2}\right)}_{/S}\cdot F$$

Finally, the equivalent volume of dihydrogen produced per metal surface unit V(H_2_)_/S_ was calculated using the ideal gas law (Equation (18)) (with P the pressure in Pa).

(18) $${V\left(H_{2}\right)}_{/S}=\frac{{n\left(H_{2}\right)}_{/S}\cdot R\cdot T}{P}$$

Figure 11a shows the corrosion rates and currents (normalised by the initial surface of beryllium), and Figure 11b shows the calculated cumulative volume of H_2_ that was produced by aqueous corrosion of Be in the different cement suspensions.

Figure 11b outlines the interest of optimising the cement chemistry to mitigate the H_2_ production due to aqueous corrosion of Be. The gas release may be decreased by a factor of 3 to 4 by replacing Portland cement with CAC, CSA0, or B40 and by a factor ≈ 50 using CSA15. Cumulative volumes of hydrogen were converted into corrosion rates using Equation (19).

(19) $${CR}_{Be}=\frac{V_{H2}\cdot M_{Be}\cdot P\cdot 365}{\rho_{Be}\cdot R\cdot T\cdot 10^{3}}$$

where V_H2_ is the cumulative H_2_ volume (in L.m^−2^·d^−1^); M_Be_ and ρ_Be_ are the molar mass and density of beryllium, respectively; and CR_Be_ is the corrosion rate of beryllium (in µm/year).

Concerning the corrosion rates, Bukaemskiy et al. [17] reported, for comparison, a corrosion rate of 0.18 ± 0.02 µm/year for Be in a NaOH solution at pH 12.6. The corrosion rates measured in PC (pH 12.7), CSA0 (pH 12.2), B40 (pH 12.1), and CAC (pH 12) suspensions are significantly higher despite similar or lower pH. Several factors may explain this discrepancy: (i) the corrosion rates are time-dependent; (ii) when Be is immersed in a cement suspension, the protective layer forming at the metal/solution interface may be abraded by the agitated cement particles; and (iii) as mentioned previously, the pH is not the only influencing factor, since the corrosion process also depends on the type and concentration of dissolved species in the aqueous solution. The smallest corrosion rate was measured in the CSA15 suspension (0.08 µm/year at 7 days—pH 11.9).

## 4. Conclusions and Outlook

This work complemented previous studies on the corrosion of Be under alkaline conditions by exploring five cementitious systems, Portland cement (reference) and four alternative binders (Portland cement blended with silica fume, calcium aluminate, and sulfoaluminate cements) selected for their reduced pore-solution pH (close to 12) as compared to the reference, which should be beneficial to Be passivation. Experiments were carried out on cement suspensions to avoid the limitation of corrosion due to lack of water that may occur in cement pastes. Be corrosion was investigated through OCP measurements, voltammetry, and EIS. The results make it possible to scale the corrosion of beryllium in all the investigated suspensions (Table 7).

The calcium sulfoaluminate cement comprising 15% anhydrite appears as the most effective in mitigating Be corrosion, followed by binary blend B40 comprising 60% Portland cement and 40% silica fume. The pore-solution pH is not the only parameter controlling the Be corrosion rate since significant differences were obtained for binders producing similar pore-solution pH values (e.g., CSA0, CAC, and B40). Future work should thus investigate more thoroughly the influence of the main ions present in the different cement pore solutions on Be corrosion. Particular attention may be brought to sulfate ions that are present at higher concentration in the two systems (CSA15 and B40) leading to the best passivation of Be. In addition, it would be necessary to extend the monitoring of Be corrosion in these two systems until thermodynamic equilibrium is reached. In suspension B40, the pozzolanic reaction was not complete and the pH (12.1) was higher than the calculated pH at thermodynamic equilibrium (11.0). Reversely, in suspension CSA15, the pH (11.9) was lower than the expected value at thermodynamic equilibrium (12.4), likely due to incomplete consumption of anhydrite. In both cases, progress of cement hydration may lead to a deviation from the optimal conditions for Be passivation. Finally, complementary work should be performed on cement pastes to investigate the corrosion of Be during the first stages of cement hydration.

## Figures and Tables

**Table 1:** Main results from the literature about aqueous corrosion of Be metal. EIS: electrochemical impedance spectroscopy.

Analytical Technique Used	Corrosive Medium	Measurement Period	Corrosion Rate	Ref.
EIS	HCl and NaOH solutions with pH ranging from 2 to 15	After 15 min of contact with the solution	Minimum at pH 12	[16]
Gravimetry	NaOH	At 10 and 20 d	- pH = 12.55: 0.18 µm/y - pH = 13.21: 1.98 µm/y - pH = 13.95: 64.4 µm/y	[17]
EIS and potentiometry	Portland, brushite, magnesium phosphate, calcium sulfoaluminate cement pastes, alkali-activated blast furnace slag	Over 150 d	Smaller in calcium sulfoaluminate cement and Portland cement pastes	[12]

**Table 2:** Properties of raw materials.

**Cement**			PC	Silica Fume	CAC	CSA Clinker	Anhydrite
Composition (wt.%)	CaO		66.70	0.67	37.40	45.07	38.25
	SiO_2_		22.40	98.10	3.92	10.55	-
	Al_2_O_3_		2.85	>0.20	50.00	23.46	-
	Fe_2_O_3_		2.25	0.51	10.58	9.70	-
	MgO		0.83	<0.20	0.17	1.00	3.14
	MnO		-	0.02	-	-	-
	Na_2_O		0.17	<0.20	-	0.17	-
	K_2_O		0.19	0.46	-	0.27	-
	Na_2_O + K_2_O		-	0.54	<0.40	-	-
	TiO_2_			<0.05	3.84	1.29	-
	P_2_O_5_		0.24	0.09	-	0.11	-
	SO_3_		2.20	0.30	0.04	8.07	52.90
	Mn_2_O_3_		-	-	-	0.01	-
	SrO		-	-	-	0.06	-
	Sulfides		0.10	<0.10	<0.10	-	-
	Chlorides		0.10	-	<0.10	0.01	-
	Ignition loss		1.70	2.21	-	0.23	-
Particle size distribution (µm)			d_10_ = 3.8 d_50_ = 15.3 d_90_ = 37.3	d_10_ = 0.5 d_50_ = 7.2 d_90_ = 13.8	d_10_ = 3.7 d_50_ = 19.6 d_90_ = 68.0	d_10_ = 3.0 d_50_ = 13.3 d_90_ = 39.0	d_10_ = 4.8 d_50_ = 18.9 d_90_ = 70.6
Specific surface area (cm^2^/g)		Blaine	3450	-	3450	4650	-
		BET	-	230,000	-	-	16,300
Crystalline phases			C_3_S (67.2%), C_2_S (17.4%), C_3_A (<5%), C_4_AF (7.1%), C$\bar{S}$ (2.7%)	Cristobalite (tr), quartz (tr), maghemite (tr)	CA (51.4%), C_2_S (8%), C_2_AS (4%), CT (3.7%), C_12_A_7_ (2.4%), C_4_AF (8%)	C_4_A_3_$\bar{S}$ (54.3%), C$\bar{S}$ (0.4%), C_2_S (29.1%), C_3_MS (4.5%), γ-F (1.0%)	C$\bar{S}$ (85%), C$\bar{S}$H_2_ (6.2%), MgCO_3_ (4.7%), CaMg(CO_3_)_2_ (4.1%)

**Table 3:** Composition and pH (measured experimentally or calculated at thermodynamic equilibrium) of the aqueous fraction of the suspensions at the end of the electrochemical tests.

	Ca (mmol/L)	Na (mmol/L)	K (mmol/L)	Si (mmol/L)	Al (mmol/L)	S (mmol/L)	pH exp. (after 7 d)	pH calc.
PC	19.9	4.6	5.0	0.01	<dL	0.03	12.7	12.7
B40	5.1	1.6	1.0	0.1	0.01	0.5	12.1	11.0
CAC	3.4	2.6	4.8	0.01	5.0	0.00	12.0	12.3
CSA0	4.2	2.8	6.4	0.01	2.4	0.02	12.2	12.5
CSA15	1.8	2.5	5.4	0.07	0.9	0.5	11.9	12.4

**Table 4:** Reduction potential of water measured experimentally and calculated using Nernst equation.

Suspension	E(H_2_O/H_2_) (V/NHE)—Measured	E(H_2_O/H_2_) (V/NHE)—Calculated
PC	−0.73	−0.76
B40	−0.64	−0.74
CAC	−0.73	−0.74
CSA0	−0.73	−0.72
CSA15	−0.70	−0.69

**Table 5:** Corrosion current at the diffusion plateau and half-wave potential measured after 3 days of immersion of Be in the different cement suspensions.

Suspension	PC	B40	CAC	CSA0	CSA15
I_corr_/S (µA/cm^2^)	312.9 ± 24.9	217.2 ± 12.5	228.4 ± 10.1	204.6 ± 17.6	115.1 ± 9.9
E_1/2_ (V/NHE)	0.02 ± 0.02	0.57 ± 0.02	0.04 ± 0.01	0.18 ± 0.02	0.59 ± 0.02

**Table 6:** Conductivity of suspensions measured experimentally and calculated from EIS after 7 d of immersion.

Suspension	Measured Conductivity (mS/cm)	Calculated Conductivity from EIS (mS/cm)
**PC**	11.2 ± 0.6	11.5 ± 1.3
**B40**	3.1 ± 0.9	2.9 ± 0.3
**CAC**	2.1 ± 0.2	2.6 ± 0.3
**CSA0**	3.3 ± 0.4	3.0 ± 0.3
**CSA15**	2.1 ± 0.1	2.4 ± 0.3

**Table 7:** Classification of the 5 cement suspensions in order of increasing corrosion of Be (1: minimum corrosion—5: maximum corrosion).

Susp.	Key Parameters (Technique Used)			
	ΔE (OCP)	J_corr_, E_1/2_ (Voltammetry)	|Z| (EIS)	CR_Be_ (EIS)
PC	2	5	5	5
B40	2	2	2	2
CAC	5	3	3	3
CSA0	2	3	3	3
CSA15	1	1	1	1

**Figure A3:**
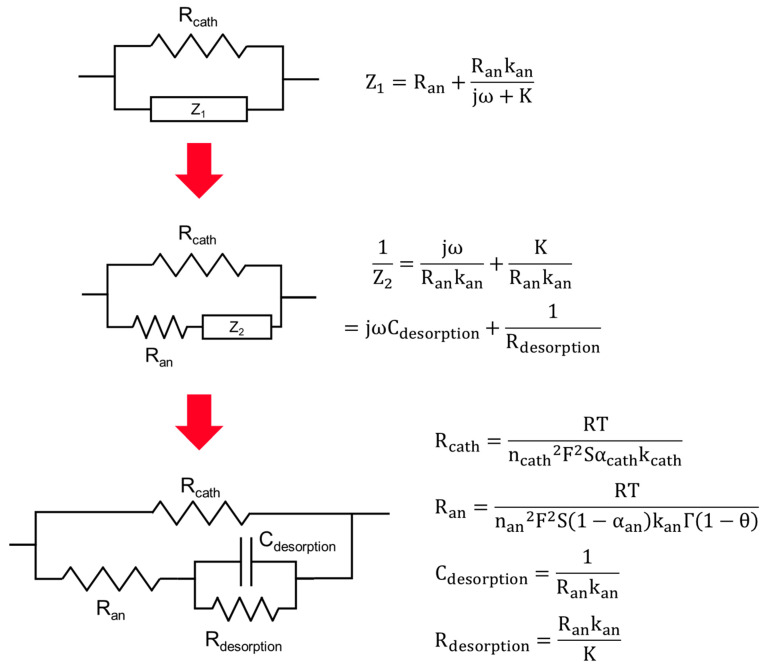
Electrical circuit and analytical expression of the electrical parameters corresponding to the Faradaic impedance associated to Be corrosion.

## Data Availability

The original contributions presented in the study are included in the article, further inquiries can be directed to the corresponding author.

## Appendix B. Definition of an Equivalent Electrical Circuit to Account for Be Corrosion

A three-step mechanism is postulated to describe beryllium corrosion:

1. Metal Be oxidation into Be(II):

(A1) $${Be}_{\left(s\right)}+\underset{\Gamma \left(1-\theta \right)}{s}\overset{k_{an}}{\to }\underset{\Gamma \theta }{{\mathrm{Be}}^{2+},s}+2e^{-}$$

(A2) $$I_{an}=n_{an}FSk_{an}\Gamma \left(1-\theta \right)$$

(A3) $$k_{an}=k_{an}^{0}{\times e}^{\left(\frac{n_{an}F}{RT}\left(1-\alpha_{an}\right)\left(E-E^{0}\right)\right)}$$

2. Reduction of water:

(A4)
$$2H_{2}O+2e^{-}\overset{k_{cath}}{\to }H_{2}+2{HO}^{-}$$

The current at the cathode is then expressed by the following relationship:

(A5) $$I_{cath}={-n}_{cath}FSk_{cath}$$

(A6) $$k_{cath}=k_{cath}^{0}{\times e}^{\left(\frac{n_{cath}F}{RT}\left(-\alpha_{cath}\right)\left(E-E^{0}\right)\right)}$$

The faradaic current I_f_ can be expressed as the sum of anodic and cathodic currents:

(A7) $$I_{f}=I_{an}+I_{cath}=n_{an}FSk_{an}\Gamma \left(1-\theta \right)-n_{cath}FSk_{cath}$$

3. Desorption (renewal of electroactive sites):

(A8)
$$\underset{\Gamma \theta }{{Be}^{2+},s}\overset{K}{\to }{\mathrm{Be}}^{2+}+\underset{\Gamma \left(1-\theta \right)}{s}$$

The variation of the number of electroactive sites as a function of time is given by

(A9) $$\Delta \theta =k_{an}\left(1-\theta \right)-K\theta =\frac{I_{an}}{n_{an}FS\Gamma }-K\theta$$

Then the faradaic can be expressed and translated into an equivalent electrical circuit as follows:

- Linearisation of I_f_:

(A10) $${\Delta I}_{f}={\left(\frac{{\partial I}_{f}}{\partial E}\right)}_{\theta }\Delta E+{\left(\frac{{\partial I}_{f}}{\partial \theta }\right)}_{E}\Delta \theta$$

(A11) $$=n_{an}FS\frac{n_{an}F}{RT}\left(1-\alpha_{an}\right)k_{an}\Gamma \left(1-\theta \right)\Delta E+n_{cath}FS\frac{n_{cath}F}{RT}\alpha_{cath}k_{cath}\Delta E-n_{an}FSk_{an}\Gamma \Delta \theta$$

- Application of Laplace transform to ΔI_f_ and Δθ:

(A12)
$$\bar{{\Delta I}_{f}}=n_{an}FS\frac{n_{an}F}{RT}\left(1-\alpha_{an}\right)k_{an}\Gamma \left(1-\theta \right)\bar{\Delta E}+n_{cath}FS\frac{n_{cath}F}{RT}\alpha_{cath}k_{cath}\bar{\Delta E}-n_{an}FSk_{an}\Gamma \bar{\Delta \theta }$$

(A13)
$$\bar{\Delta \theta }=\frac{k_{an}\frac{n_{an}F}{RT}\left(1-\alpha_{an}\right)\left(1-\theta \right)}{j\omega +k_{an}+K}\bar{\Delta E}$$

- We pose:

(A14)
$$\frac{1}{R_{an}}=n_{an}FS\frac{n_{an}F}{RT}\left(1-\alpha_{an}\right)k_{an}\Gamma \left(1-\theta \right)$$

(A15)
$$\frac{1}{R_{cath}}=n_{cath}FS\frac{n_{cath}F}{RT}\alpha_{cath}k_{cath}$$

- We obtain:

$$\bar{{\Delta I}_{f}}=\left(\frac{1}{R_{an}}+\frac{1}{R_{cath}}\right)\bar{\Delta E}-\left(n_{an}FSk_{an}\Gamma \frac{k_{an}\frac{n_{an}F}{RT}\left(1-\alpha_{an}\right)\left(1-\theta \right)}{j\omega +k_{an}+K}\right)\bar{\Delta E}$$

(A16)
$$=\left(\frac{1}{R_{an}}+\frac{1}{R_{cath}}\right)\bar{\Delta E}-\left(\frac{1}{R_{an}}\times \frac{k_{an}}{j\omega +k_{an}+K}\right)\bar{\Delta E}$$

- As Z_f_ = ΔE/ΔI_f_, we finally obtain:

(A17)
$$\frac{1}{Z_{f}}=\frac{1}{R_{an}}+\frac{1}{R_{cath}}-\frac{k_{an}}{R_{an}\left(j\omega +k_{an}+K\right)}$$

## Appendix C. Electrical Parameters Used to Fit the EIS Spectra of Be

**Table A1:** Set of electrical parameters used to fit the EIS spectra recorded on Be electrodes in the different cement suspensions.

Susp.	Time (d)	Self (µH·cm^2^)	R_electrolyte_ (Ω·cm^2^)	C_double layer_ (µF/cm^2^)	α	R_charge transfer_ (Ω·cm^2^)
PC	0	5.0 ± 1.0	18.0 ± 2.0	13.0 ± 2.0	0.91 ± 0.01	3.25 ± 0.04 × 10^4^
	1	5.0 ± 1.0	18.0 ± 2.0	13.0 ± 2.0	0.91 ± 0.01	3.25 ± 0.04 × 10^4^
	2	5.0 ± 1.0	18.0 ± 2.0	11.0 ± 1.0	0.91 ± 0.01	4.75 ± 0.04 × 10^4^
	3	5.0 ± 1.0	18.0 ± 2.0	11.0 ± 1.0	0.91 ± 0.01	5.80 ± 0.20 × 10^4^
	7	5.0 ± 1.0	20.0 ± 2.0	11.0 ± 1.0	0.91 ± 0.01	6.80 ± 0.04 × 10^4^
B40	0	13.0 ± 4.0	55.0 ± 10.0	7.0 ± 1.0	0.90 ± 0.01	1.50 ± 0.01 × 10^5^
	1	13.0 ± 4.0	60.0 ± 10.0	8.0 ± 0.3	0.90 ± 0.01	1.47 ± 0.06 × 10^5^
	2	13.0 ± 4.0	65.0 ± 10.0	8.5 ± 0.5	0.90 ± 0.01	1.62 ± 0.02 × 10^5^
	3	14.0 ± 4.0	70.0 ± 10.0	8.0 ± 0.5	0.90 ± 0.01	2.03 ± 0.03 × 10^5^
	7	14.0 ± 4.0	95.0 ± 10.0	9.3 ± 0.5	0.85 ± 0.01	2.80 ± 0.05 × 10^5^
CAC	0	24.0 ± 10.0	86.0 ± 20.0	7.5 ± 1.0	0.93 ± 0.02	1.93 ± 0.03 × 10^5^
	1	24.0 ± 10.0	80.0 ± 20.0	10.0 ± 1.0	0.93 ± 0.02	1.73 ± 0.03 × 10^5^
	2	24.0 ± 10.0	90.0 ± 15.0	10.0 ± 1.0	0.93 ± 0.02	1.50 ± 0.03 × 10^5^
	3	24.0 ± 10.0	90.0 ± 10.0	10.5 ± 0.5	0.93 ± 0.02	1.65 ± 0.03 × 10^5^
	7	24.0 ± 10.0	95.0 ± 10.0	11.0 ± 0.5	0.91 ± 0.02	1.60 ± 0.03 × 10^5^
CSA0	0	24.0 ± 5.0	87.0 ± 10.0	10.0 ± 1.0	0.88 ± 0.01	1.10 ± 0.02 × 10^5^
	1	24.0 ± 5.0	90.0 ± 10.0	10.0 ± 5.0	0.91 ± 0.02	1.37 ± 0.02 × 10^5^
	2	24.0 ± 5.0	90.0 ± 10.0	11.0 ± 5.0	0.89 ± 0.02	1.42 ± 0.02 × 10^5^
	3	24.0 ± 5.0	85.0 ± 10.0	11.0 ± 5.0	0.88 ± 0.02	1.78 ± 0.02 × 10^5^
	7	24.0 ± 5.0	90.0 ± 10.0	11.8 ± 5.0	0.85 ± 0.02	1.75 ± 0.02 × 10^5^
CSA15	0	0.10 ± 0.05	90.0 ± 10.0	4.8 ± 0.2	0.87 ± 0.02	1.85 ± 0.2 × 10^6^
	3	1.00 ± 0.03	71.3 ± 5.0	6.7 ± 0.3	0.91 ± 0.02	3.00 ± 0.5 × 10^6^
	4	1.00 ± 0.05	65.7 ± 5.0	6.2 ± 0.3	0.92 ± 0.01	3.20 ± 0.6 × 10^6^
	5	1.00 ± 0.05	67.7 ± 5.0	6.3 ± 0.3	0.92 ± 0.02	3.10 ± 0.6 × 10^6^
	6	1.00 ± 1.00	63.5 ± 5.0	6.7 ± 0.3	0.91 ± 0.02	2.70 ± 0.5 × 10^6^
	7	1.50 ± 0.50	63.0 ± 5.0	6.85 ± 0.30	0.91 ± 0.02	2.30 ± 0.5 × 10^6^

## Appendix D. List of Principal Symbols

L_t_[E(t)], L_t_[I(t)]	Laplace transform applied to the potential and the current
Z	Impedance (Ω)
Z_real_, Z_imag_	Real and imaginary parts of the impedance (Ω)
|Z|, ϕ	Modulus (Ω) and phase (°) of the impedance
E(H^+^/H_2_)	Reduction potential of water (V/ENH)
E^0^(H^+^/H_2_)	Standard reduction potential of water (V/ENH)
F	Faraday constant (C)
R	Ideal gas constant (J/K/mol)
P	Pressure (Pa)
s	Electroactive site
Γ	Electroactive sites number
θ	Proportion of occupied sites
k_an_, k_cath_	Anodic and cathodic kinetic charge transfer constants (s^−1^)
K	Kinetic desorption charge transfer constant (s^−1^)
n_an_, n_cath_	Number of electrons taking part into the anodic and cathodic charge transfer reactions
S	Surface of electrode/suspension interface (cm^2^)
k^0^_an_, k^0^_cath_	Anodic and cathodic intrinsic transfer constants
α_an_, α_cath_	Anodic and cathodic charge transfer constants
Q_/S_	Charge (C/cm^2^)
I_corr_/S = J_corr_	Corrosion current normalised by the reactive surface of beryllium (µA/cm^2^, nA/cm^2^)
E_1/2_	Half-wave potential (V/NHE)
R_electrolyte_	Electrolyte resistance (Ω/cm^2^)
C_double layer_	Double-layer capacitance (F/cm^2^)
α	Non-ideality coefficient associated to the CPE
R_charge transfer_	Charge transfer resistance (Ω/cm^2^)
γ	Electrical conductivity (mS/cm)
k	Parameter associated to the geometry of the electrochemical cells (cm^−1^)
n_e_	Number of electrons exchanged
n(H_2_)_/S_	Number of moles of hydrogen produced normalised by the reactive surface (mol/cm^2^)
V(H_2_)_/S_	Hydrogen volume produced normalised by the reactive surface (mol/cm^2^)
CR_Be_	Corrosion rate of beryllium (µm/year)
M_Be_	Molar mass of beryllium (g/mol)
ρ_Be_	Density of beryllium (g/cm^3^)
